# Nutritional improvement status of primary and secondary school students in the pilot nutrition improvement areas of Hainan, China from 2014 to 2021

**DOI:** 10.1186/s12887-024-04910-z

**Published:** 2024-07-11

**Authors:** Diyue Liu, Fan Zhang, Yanming Zhang, Yuting Wu, Jingjing Lu, Chunbo Dong, Yingchen Xiao, Xiaoyu Xiao, Jiaqing Zhang, Qiqin Feng

**Affiliations:** 1 School of Public Health, Hainan Medical University, No.3 Xueyuan Road, Longhua District, Haikou City, China; 2 Faculty of Medicine, Macau University of Science and Technology, Macau, China; 3 School of Public Health, North China University of Science and Technology, Tangshan, China; 4 Danzhou Center for Diseases Prevention and Control, Danzhou, China; 5 School of Public Health, Tianjin Medical University, Tianjin, China; 6 Postgraduate Department, Hainan Medical University, Haikou, China

**Keywords:** Student Nutrition Improvement Program, Primary and secondary school students, Nutritional status, Changing trends

## Abstract

**Objective:**

By meticulously tracking the evolving growth, development, and nutritional status of primary and secondary school students in Qiongzhong County from 2014 to 2021 post-implementation of the “Nutrition Improvement Program for Rural Compulsory Education Students”(NIPRCES, This project provides a supplementary food allowance of at least ￥4 per person per day for primary and secondary school students. The project area undergoes annual routine monitoring.), this study aims to offer a scientific basis for enhancing and promoting the project. Through thorough monitoring of students’ nutritional status changes influenced by this program, we strive to establish a comprehensive and evidence-based framework for its future advancement.

**Methods:**

From 2014 to 2021, this study employed a multi-stage sampling method utilizing cluster sampling to select six primary and six secondary schools in Qiongzhong County, Hainan Province. Data on the growth and development of respondents were collected. This cohort was a dynamic cohort with a total of 18,762 final data recovered. The prevalence of malnutrition was evaluated using the Cochran Armitage Trend Test (CATT) to assess year-to-year changes. Furthermore, height/weight and the prevalence of malnutrition between groups were compared using the t-test, χ2 test, and Bonferroni’s corrected analysis.

**Results:**

The average height of both boys and girls has increased. In 2021, boys and girls of all ages showed an average height increase of 2.31 cm and 1.98 cm, respectively, compared to 2014. Nevertheless, the growth and development levels, and rate of improvement of these students remain comparatively lower than their rural counterparts across China, who are of the same age. From 2014 to 2021, the prevalence of undernutrition (mainly wasting) showed a significant downward trend (*P* < 0.05) from 29.30% to 22.19%, and the prevalence of overnutrition showed an upward trend (both *P* < 0.05). The prevalence of undernutrition was higher among boys, students in grades 1–3 and those of Li nationality. Meanwhile, the prevalence of overnutrition was higher among boys, students in grades 1–3 and those of Han nationality.

**Conclusions:**

Over the 8-year period of NIPRCES, there has been progress in the growth and development of students, yet levels still lag behind the national average for rural students of the same age. While malnutrition prevalence have decreased, they remain high, with a concerning rise in overnutrition prevalence. Undernutrition and overweight/obesity are more prevalent among boys and younger students. Li students show higher prevalence of undernutrition, while overnutrition is a growing issue among Han students. Simultaneously, local education and health care departments must acknowledge the disparities in growth and nutritional status among primary and secondary school students residing in rural areas within the tropics and those in rural areas across the entire nation. Nutritional improvement measures should be tailored to local conditions.

## Introduction

Childhood and adolescence are the key stages of human development. A good nutrition is an essential for their growth, development, physical and mental health [1, 2] Good nutritional status even has a profound positive impact on their lifelong health [3]. Conversely, malnutrition in childhood (including undernutrition and overnutrition) not only severely hampers the growth and brain development in children and adolescents, but also makes them more susceptible to metabolic syndrome in adulthood [1]. Malnutrition in childhood also negatively affects individual learning ability, cognitive ability, and mental health [4].Malnutrition is a widespread public health problem worldwide, especially among children and adolescents, and the World Health Organization (WHO) data in 2019 showed that more than 17% of children and adolescents worldwide were overweight and obese in 2016. As of 2017, 51 million children around the world were wasting [2]. The data from《the Blue Book for Children: China’s Child Development Report (2021) 》showed that as of 2019, 8.5% of children in China were undernourished, and the prevalences of overweight or obese children were even 24.2% [3].

China is a vast country, and different regions have significant differences in terms of economy, culture, and living habits, which can affect the nutritional status of children [5, 6]. Several studies demonstrated [7, 8]that undernutrition prevalence among children in economically underdeveloped areas was higher than that in economically developed areas. In order to improve the nutritional status of Chinese primary and secondary school students, the Chinese government has implemented policies such as the “Chinese Students Drinking Milk Plan” [9]. Yanhui Dong’s survey [10] on the prevalence of undernutrition among urban and rural children in 31 provinces in China has showed that the gap in the prevalence of undernutrition between urban and rural children in China has gradually narrowed with the promotion and support of national policies. But there are still some differences between eastern and western provinces, and the gap between urban and rural areas still exists. To improve the nutritional status of primary and secondary school students in rural areas of China and narrow the urban–rural gap, the Chinese government launched the “Nutrition Improvement Program for Rural Compulsory Education Students” (NIPRCES) in November 2011 in rural areas of 22 provinces in central and western China [11].

This study aims to investigate the growth and development trends, as well as the nutritional status trends, of compulsory education students over the eight-year period of NIPRCES implementation in Qiongzhong County. Initially, the study seeks to assess changes in the height and weight of students in this area compared to national trends over the same period through trend descriptions. Subsequently, the study intends to analyze trends in the nutritional status of students within the site. Lastly, the study aims to analyze the trend of nutritional status improvement among respondents from diverse demographic backgrounds over the eight-year period, providing theoretical underpinning for enhanced NIPRCES implementation in the region.

## Materials and Methods

### Study subjects

All the monitoring in this study was conducted by the 《Work Manual for Monitoring and Evaluation of Nutritional Health Status of Students under NIPRCES》. In Qiongzhong County, where the NIPRCES has been implemented, the stratified random cluster sampling method was used to obtain the sample. The researchers took no less than 10% of the total from local primary and secondary schools as monitoring schools, respectively. Then, at least 40 students from one or two classes in each grade, from the first grade of primary school to the third grade of secondary school, were selected as the respondents, with a basic balance of boys and girls. After the monitoring classes and students were determined, they have been kept relatively fixed during the monitoring period, in other words, the monitoring classes have been kept basically unchanged, but some monitoring students would be lost due to promotion, school transfer, class transfer, sick leave, etc. Meanwhile, some new students were added. This study is derived from the NIPRCES monitoring program, which has been in existence for a long time along with the nutrition improvement policy, some of the results have supported the writing and publication of the relevant dissertations [12], and only some of the variables were included in the study, and the current monitoring of NIPRCES is still ongoing.

**Fig. 1:**
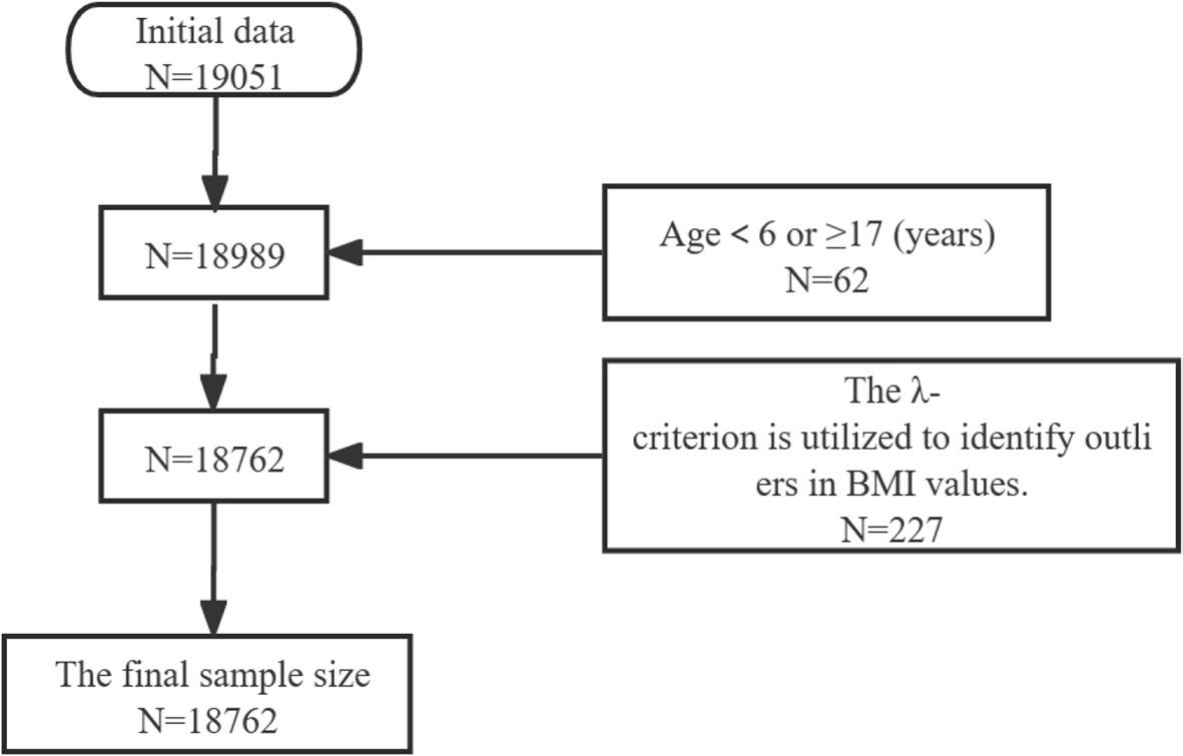
Flowchart for Sample Size Screening

Following the sampling principles outlined above, a total of 18,762 individuals have been included in this study from the commencement of monitoring in 2014 through 2021. The sample screening process is elaborated in Fig. 1.

### Study Design

During the annual monitoring process, interviewed students and their parents will collaborate with the program team to fill out a personal information(such as: gender, age, grade level, and ethnicity) questionnaire after completing the informed consent form. Additionally, the students will undergo a physical examination to measure their height and weight. Informed consent was obtained from the student and the student’s parent or legal guardian before the student was included in the study. The NIPCRES was approved by the Ethics Committee of Hainan Medical University (HYLL-2023–486). Participant information was anonymized and de-identified prior to analysis. The investigation and analysis were conducted in accordance with the principles outlined in the Declaration of Helsinki.

This study referred to the《Technical Specification for Student Health Examination (GB/T 26343–2010)》 [13]. The height and weight of the respondents were measured, and the body mass index (BMI) was calculated as follows: BMI = weight/height^2^ (kg/m^2^). The baseline survey about the height and weight of the subjects was conducted in March 2014, followed by monitoring in November and December each year.

Screening for undernutrition among the subjects was based on the “Screening standard for malnutrition of school-age children and adolescents (WS/T456-2014)” [14], and Screening for overweight and obesity among them was based on “Screening for overweight and obesity among school-age children and adolescents (WS/T 586–2018)” [15]. In this study, participants were divided by age and evaluated for their height and BMI. The analysis involved assessing if their height met the national standard for stunting and determining if their BMI indicated wasting, overweight, or obesity. Individuals whose measurements did not align with these criteria were classified as having a normal nutritional status. Furthermore, participants were categorized as experiencing undernutrition if they were identified as stunted or wasted, and as experiencing overnutrition if they were classified as overweight or obese.

### Nutrition improvement program for rural compulsory education students

After the national NIPRCES program was implemented in 2011, Hainan Province, which is an underdeveloped province located in southern China and the only province in the tropics, initiated a pilot program in 2013 in Qiongzhong County and other impoverished areas of Hainan Province. To improve the nutritional health of students in poor rural areas, China began giving meal subsidies of 4 yuan per person per day to poor cities and counties nationwide in late 2014. At first, the project was only carried out in the pilot poor cities and counties, and later the coverage of the project gradually increased. In this study, Qiongzhong County was one of the earliest pilot areas. The district's meal subsidy is used exclusively for centralized purchasing of ingredients for meals in school cafeterias. NIPRCES not only provides grants for enhanced nutrition but also regularly monitors districts that benefit from these nutritional enhancements. The data for this study is derived from the routine monitoring conducted by NIPRCES.

To guarantee that the student meals offered through NIPRCES are clean, nutritious, and consumed by students, the Chinese government introduced the “Implementation Rules of the Nutrition Improvement Program for Rural Compulsory Education Students” as early as 2012 [16]. These guidelines aim to effectively enhance student nutrition at the policy level. The local government sets up school canteens and includes meal subsidies in the canteen services to ensure that every student has access to nutritional support for improvement.

### Statistical analysis

The data were entered in Excel 2010 and imported into SPSS 22.0 statistical program for analysis. The quantitative data were expressed using the mean ± standard deviations, and the qualitative data was represented by rate or composition ratio. Differences in height and weight between years were examined using t-tests. The χ^2^ test was used for comparison between two groups, and the Bonferroni method was used for multiple comparisons based on the χ^2^ test for comparison between multiple groups. The Cochran Armitage trend test (CATT) was used for the trend analysis of nutritional status from 2014 to 2021. In this study, the test level α was set at 0.05, and when *P* < 0.05 indicated that the difference was statistically significant.

### Quality control

The height and weight of the respondents in this study were measured by professionals using uniform instruments (height measuring device and weight scale). All the measuring instruments were calibrated and zeroed before use, and the measurements were registered in the record sheets to one decimal place.

All data were double-entered in Epidata 3.1 software. Abnormal data were screened according to the cleaning principle. Once abnormal values were found, they should be returned to the original record sheet for verification and correction. If the original data in record sheets were still abnormal, clearing the abnormal data according to the λ guidelines, and backing up the original database and cleaned database.

## Results

### Basic information on primary and secondary school students from 2014 to 2021

Students in the 16 years and older age group were not included in the analysis because the number of students in these age groups was too small to be representative of the overall situation in this age group. A total of 18,762 students were included in this study, and the number of students included from 2014 to 2021 was 1951, 1799, 2399, 2201, 2358, 2590, 2810, and 2706, respectively, with a balanced ratio of boys to girls in all years, and the respondents were dominated by Li students. For details, see Table 1.

**Table 1:** General demographic characteristics of participants (*N* = 18,762 visits)

Years	Sex		Grade			Nation			Total
	Boys	Girls	1 ~ 3	4 ~ 6	7 ~ 9	Han	Li	Other ethnic minorities	
2014	937(48.9)	978(51.1)	564(29.5)	763(39.8)	588(30.7)	452(23.6)	1233(64.4)	230(12.0)	1915(10.1)
2015	939(52.2)	860(47.8)	512(28.5)	708(39.4)	579(32.2)	411(22.9)	1162(64.6)	226(12.6)	1799(9.6)
2016	1236(51.5)	1163(48.5)	641(26.7)	962(40.1)	796(33.2)	470(19.6)	1669(69.6)	260(10.8)	2399(12.8)
2017	1134(51.5)	1067(48.5)	644(29.3)	744(33.8)	813(36.9)	387(17.6)	1567(71.2)	247(11.2)	2201(11.7)
2018	1176(49.9)	1182(50.1)	713(30.2)	837(35.5)	808(34.3)	440(18.7)	1690(71.7)	228(9.7)	2358(12.5)
2019	1398(54.0)	1192(46.0)	799(30.9)	898(34.7)	893(34.5)	418(16.1)	1930(74.5)	242(9.3)	2590(13.8)
2020	1491(53.1)	1318(46.9)	868(30.9)	1065(37.9)	876(31.2)	492(17.5)	2044(72.8)	273(9.7)	2809(15.0)
2021	1391(51.7)	1300(48.3)	801(29.8)	964(35.8)	926(34.4)	507(18.8)	1979(73.5)	205(7.6)	2691(14.3)
Total	9702(51.7)	9060(48.3)	5542(29.8)	6941(37.0)	6279(33.5)	3577(19.1)	13,274(70.6)	1911(10.2)	18,762(100.0)

The number to the left of () is the example number, and the number in () is the percentage

### Primary and secondary school student’s height and weight status and change trend from 2014 to 2021

**Fig. 2:**
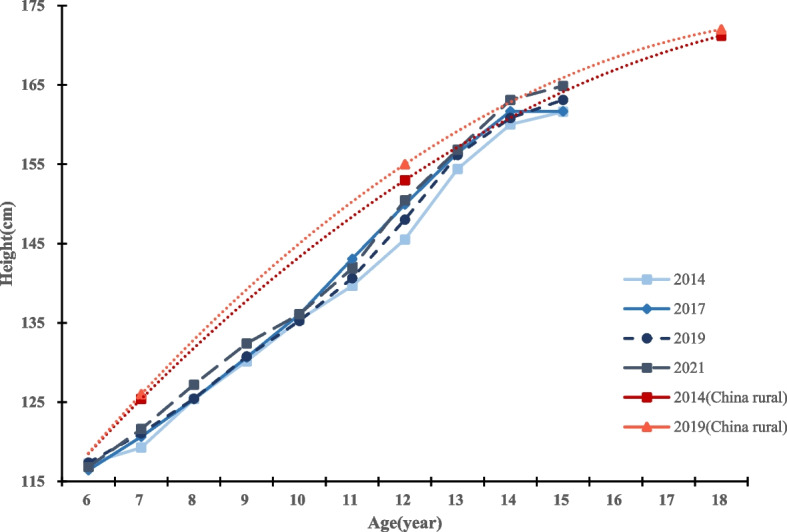
Height levels and trends of boys in different age groups in different years

**Fig. 3:**
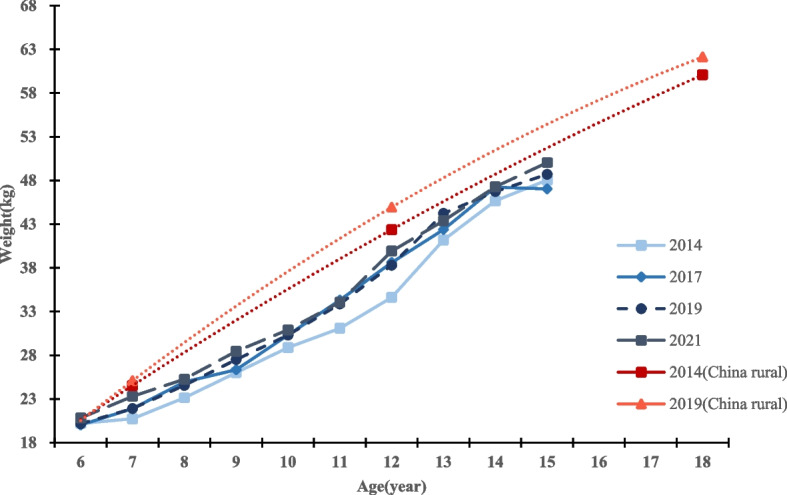
Weight levels and trends of boys in different age groups in different years

From 2014 to 2021, the average height of both boys and girls in Qiongzhong County showed an increasing trend. Eight years after the implementation of the NIPRCES, the average height of boys and girls increased from 138.87 cm to 141.18 cm (growth of 2.31 cm) and from 137.97 cm to 139.95 cm (growth of 1.98 cm), respectively. Among them, the height growth was significant in both boys and girls 7 ~ 9 years old groups and 13 ~ 14 years old groups (*P* value < 0.05). 6 years old group students showed negative height growth, but the difference was not statistically significant. From 2014 to 2017 (the first 4 years), the height growth rates of boys and girls were 0.33 cm/year and 0.27 cm/year, respectively; from 2017 to 2021 (the last 4 years), the height growth rates of boys and girls were 0.25 cm/year and 0.22 cm/year, respectively. The level of growth and development exhibited by boys in this region is comparatively lower than that observed among boys in rural China within the same timeframe. For details, see Tables 2 and 3, and Figs. 2 and 3. The data on growth and development levels of rural students nationwide in Figs. 1 and 2 are derived from data in the published article by Dongmei Luo et al. [17].

**Table 2:** Trends in height and weight of boys in the Qiongzhong County, 2014–2021

Age	2017–2014				2021–2017				2021–2014			
	Height(cm)	*t*	Weight(cm)	*t*	Height(kg)	*t*	Weight(kg)	*t*	Height(cm)	*t*	Weight(kg)	*t*
6	-0.8	-0.5	-0.2	-0.2	0.2	0.2	0.5	0.9	-0.7	-0.5	0.3	0.3
7	1.4	1.9	1.2	2.3^**^	1.0	1.5	1.5	2.3^**^	2.4	3.4^**^	2.6	4.4^***^
8	0.0	0.0	1.8	2.7^**^	1.8	2.4^**^	0.3	0.5	1.8	2.4^**^	2.1	3.9^***^
9	0.5	0.5	0.3	0.5	1.7	2.4^**^	2.0	3.0^**^	2.2	2.6^**^	2.3	3.0^**^
10	0.7	0.8	1.4	1.6	-0.1	-0.1	0.4	0.5	0.6	0.8	1.9	2.4^**^
11	3.4	3.3^**^	3.2	3.4^**^	-1.2	-1.2	-0.2	-0.3	2.2	2.5^**^	3.0	3.5^**^
12	4.4	4.9^***^	4.0	4.5^***^	0.4	0.4	0.8	0.8	4.8	4.8^***^	4.8	4.9^***^
13	2.0	1.8	1.2	1.0	0.3	0.3	0.7	0.7	2.3	2.2^**^	1.9	1.8
14	1.7	1.4	1.6	1.2	1.9	1.7	0.3	0.4	3.5	3.6^***^	1.8	1.6
15	0.1	0.1	-1.0	-0.6	3.8	3.0^**^	3.1	2.0	3.9	3.4^**^	2.1	1.3
Mean	1.3		1.4		1.0		0.9		2.3		2.3	

*P < 0.05, **P < 0.01, ***P < *0.001*

**Table 3:** Trends in height and weight of girls in the Qiongzhong County, 2014–2021

Age	2017–2014				2021–2017				2021–2014			
	Height(cm)	*t*	Weight(cm)	*t*	Height(kg)	*t*	Weight(kg)	*t*	Height(cm)	*t*	Weight(kg)	*t*
6	-0.5	-0.4	0.8	1.4	-1.4	-1.8	-0.4	-0.8	-1.9	-1.6	0.4	0.6
7	0.9	1.2	0.8	1.8	2.0	2.6^**^	2.0	4.0^***^	2.9	3.7^***^	2.9	6.0^***^
8	-1.6	-1.9	0.8	1.4	3.4	4.2^***^	1.7	2.9^**^	1.8	2.3^**^	2.6	4.4^***^
9	1.1	1.3	1.9	3.2^**^	1.8	2.3^**^	0.9	1.5	3.0	3.6^***^	2.8	4.6^***^
10	2.1	2.2^**^	2.3	3.1^**^	1.6	2.0	1.3	1.8	3.6	4.3^***^	3.6	5.1^***^
11	1.0	1.1	1.1	1.3	0.3	0.3	-0.5	-0.6	1.2	1.3	0.6	0.7
12	3.2	4.1^***^	3.2	4.3^***^	-0.9	-1.2	-0.5	-0.7	2.3	2.5^**^	2.7	3.2^**^
13	2.8	4.0^***^	2.7	3.7^***^	-0.4	-0.7	-1.2	-1.7	2.3	3.5^***^	1.5	2.3^**^
14	1.8	2.4^**^	1.4	1.6	1.1	1.5	1.0	1.2	2.9	4.2^***^	2.4	2.8^**^
15	0.1	0.1	0.1	0.1	1.6	1.4	0.9	0.7	1.7	1.8	1.0	1.0
Mean	1.1		1.5		0.9		0.6		2.0		2.1	

*P < 0.05, **P < 0.01, ***P < *0.001*

**Fig. 4:**
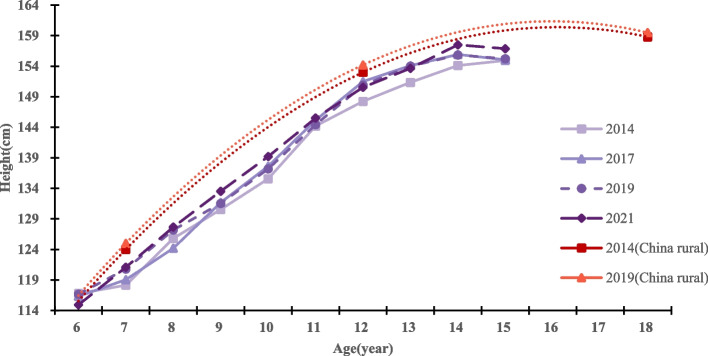
Height levels and trends of girls in different age groups in different years

**Fig. 5:**
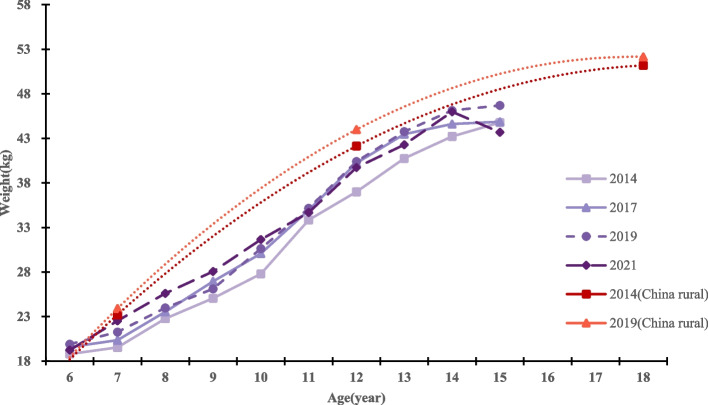
Weight levels and trends of girls in different age groups in different year

From 2014 to 2021, the average weight of primary and secondary school students in Qiongzhong County showed an increasing trend, with the average weight of boys and girls increasing from 31.95 kg to 34.24 kg (growth of 2.29 kg) and from 31.36 kg to 33.42 kg (growth of 2.06 kg), respectively. All age groups of boys and girls showed positive weight gain, with significant weight gain in the 7 ~ 10 year old group and 12 year old group for both boys and girls (all *P* < 0.05). From 2014 to 2017 (the first 4 years), the weight growth rates of boys and girls were 0.34 kg/year and 0.38 kg/year, respectively. From 2017 to 2021 (the last 4 years), the weight growth rates of boys and girls were 0.23 kg/year and 0.14 kg/year, respectively. The level of growth and development exhibited by boys in this region is comparatively lower than that observed among girls in rural China within the same timeframe. For details, see Tables 2 and 3, and Figs. 4 and 5. The data on growth and development levels of rural students nationwide in Figs. 3 and 4 are derived from data in the published article by Dongmei Luo et al. [17].

### Primary and secondary school students’ nutrition status and change trend from 2014 to 2021

In this study, undernutrition was divided into stunting and emaciation for analysis. From 2014 to 2021, the prevalence of undernutrition among students showed a significant decrease (*P* < 0.05), decreasing by 7.11%, with the prevalences of stunting and emaciation decreasing by 2.79% and 4.32%, respectively (both *P* < 0.05). However, during the 8 years of the NIPRCES, the prevalences of overweight and obesity both showed an increasing trend (both* P* < 0.05) by 2.86% and 2.74%, respectively. For details, see Table 4.

**Table 4:** Student nutritional status and changes in Qiongzhong County, 2014–2021

Years		Undernutrition						Overweight		Obesity	
		Stunting		Wasting		Total					
	*N*	*n*	%	*n*	%	*n*	%	*n*	%	*n*	%
2014	1915	99	5.2	462	24.1	561	29.3	47	2.5	25	1.3
2015	1799	113	6.3	378	21.0	491	27.3	62	3.5	42	2.3
2016	2399	130	5.4	489	20.4	619	25.8	109	4.5	57	2.4
2017	2201	77	3.5	454	20.6	531	24.1	110	5.0	59	2.7
2018	2358	82	3.5	512	21.7	594	25.2	105	4.5	63	2.7
2019	2590	87	3.4	496	19.2	583	22.5	146	5.6	70	2.7
2020	2809	93	3.3	595	21.2	688	24.5	124	4.4	100	3.6
*2021*	2691	64	2.4	533	19.8	597	22.2	143	5.3	109	4.1
*χ*^*2*^_*CATT*_			**57.1**		**7.3**		**35.5**		**20.5**		**35.3**
*P*			** < 0.001**		**0.007**		** < 0.001**		** < 0.001**		** < 0.001**

### Nutritional status and changing trends of primary and secondary school students by sex from 2014 to 2021

From 2014 to 2021, the prevalence of undernutrition for both boys and girls showed a decreasing trend (both *P* < 0.05), decreasing by 8.94% and 5.85%, respectively. Among them, the prevalence of stunting decreased by 3.35% for boys and 2.27% for girls, the prevalence of emaciation decreased by 5.59% for boys and 3.58% for girls, all of which showed a decreasing trend (all *P* < 0.05). The prevalence of overweight and obesity for both boys and girls showed an increasing trend during the 8 years (all *P* < 0.05), among which the prevalence of overweight increased by 3.12% for boys and 2.57% for girls, and the prevalence of obesity increased by 3.15% for boys and 2.24% for girls.

The prevalence of undernutrition was higher in boys than in girls during all 8 years (all *P* < 0.05). The prevalence of stunting was higher in boys than in girls except in 2014 and 2021 (all *P* < 0.05), while the prevalence of emaciation was higher in boys than in girls in all years (all *P* < 0.05). The prevalence of overweight was higher in boys than in girls, but the differences between them were statistically significant only in 2015 and 2019 (both *P* < 0.05). The prevalence of obesity was also higher in boys than in girls, and the differences between them were statistically significant in all years except 2015 (all *P* < 0.05). For details, see Table 5.

**Table 5:** Nutritional status of primary and secondary school students by sex, 2014~2021 [*n*(%)]

Years			2014	2015	2016	2017	2018	2019	2020	2021	*χ*^2^_CATT_	*P*
Undernutrition	Stunting	Boys	55(5.9)	71(7.6)	84(6.8)	50(4.4)	53(4.5)	57(4.1)	62(4.2)	35(2.5)	**36.6**	**<0.001**
		Girls	44(4.5)	42(4.9)	46(4.0)	27(2.5)	29(2.5)	30(2.5)	31(2.4)	29(2.2)	**22.2**	**<0.001**
		*χ*^2^	1.8	**5.5**	**9.4**	**5.8**	**7.4**	**4.8**	**7.1**	0.2		
		*P*	0.2	**0.02**	**0.002**	**0.02**	**0.007**	**0.03**	**0.01**	0.6		
	Wasting	Boys	278(29.7)	237(25.2)	292(23.6)	289(25.5)	322(27.4)	327(23.4)	396(26.6)	335(24.1)	2.7	0.10
		Girls	184(18.8)	141(16.4)	197(16.9)	165(15.5)	190(16.1)	169(14.2)	199(15.1)	198(15.2)	**7.3**	**0.007**
		*χ*^2^	**30.8**	**21.2**	**16.5**	**33.7**	**44.3**	**35.3**	**55.0**	**33.2**		
		*P*	**<0.001**	**<0.001**	**0.001**	**<0.001**	**<0.001**	**<0.001**	**<0.001**	**<0.001**		
	Total	Boys	333(35.5)	308(32.8)	376(30.4)	339(29.9)	375(31.9)	384(27.5)	458(30.7)	370(26.6)	**19.2**	**<0.001**
		Girls	228(23.3)	183(21.3)	243(20.9)	192(18.0)	219(18.5)	199(16.7)	230(17.5)	227(17.5)	**21.1**	**<0.001**
		*χ*^2^	**34.5**	**30.0**	**28.4**	**42.5**	**55.8**	**42.8**	**66.6**	**32.5**		
		*P*	**<0.001**	**<0.001**	**<0.001**	**<0.001**	**<0.001**	**<0.001**	**<0.001**	**<0.001**		
Overweight		Boys	24(2.6)	42(4.5)	65(5.3)	65(5.7)	59(5.0)	94(6.7)	76(5.1)	79(5.7)	**9.3**	**0.002**
		Girls	23(2.4)	20(2.3)	44(3.8)	45(4.2)	46(3.9)	52(4.4)	48(3.6)	64(4.9)	**11.0**	**0.001**
		*χ*^2^	0.1	**6.2**	3.0	2.7	1.75	**6.8**	3.5	0.76		
		*P*	0.8	**0.01**	0.1	0.1	0.2	**0.009**	0.1	0.4		
Obesity		Boys	19(2.0)	26(2.8)	40(3.2)	41(3.6)	41(3.5)	50(3.6)	69(4.6)	72(5.3)	**20.4**	**<0.001**
		Girls	6(0.6)	16(1.9)	17(1.5)	18(1.7)	22(1.9)	20(1.7)	31(2.4)	37(2.9)	**14.0**	**<0.001**
		*χ*^2^	**7.4**	1.6	**8.1**	**7.8**	**6.0**	**8.8**	**10.6**	**58.7**		
		*P*	**0.006**	0.20	**0.004**	**0.005**	**0.01**	**0.003**	**0.001**	**0.002**		

### Nutritional status and changing trends of primary and secondary school students by grades from 2014 to 2021

From 2014 to 2021, the prevalence of undernutrition among students in grades 1 ~ 3, 4 ~ 6, and 7 ~ 9 showed a decreasing trend (all *P* < 0.05), decreasing by 10.73%, 7.03%, and 3.45%, respectively. Among them, the prevalence of stunting decreased by 3.20%, 3.43%, and 1.47% for the grades 1 ~ 3, 4 ~ 6, and 7 ~ 9, respectively, with a decreasing trend (all *P* < 0.05). And the prevalence of emaciation decreased by 7.53% in grades 1 ~ 3, with a decreasing trend (*P* < 0.05), but the decreasing trend of the prevalence of emaciation was not significant in grades 4 ~ 6 and 7 ~ 9. The prevalences of overweight and obesity among students in grades 1 ~ 3 increased by 5.60% and 4.94%, respectively, and the prevalence of obesity among students in grades 4 ~ 6 increased by 4.32%, all showing a significant upward trend (all *P* < 0.05). However, the rising trend of the prevalences of overweight and obesity among students in grades 7 ~ 9 was not significant.

Over the 8 years, the prevalence of undernutrition was higher in grades 1 ~ 3 than in grades 7 ~ 9 except in 2015, 2019 and 2021 (all *P* < 0.017). Among which the prevalence of stunting was higher in grades 1 ~ 3 than in grades 7 ~ 9 (all *P* < 0.017), and in the prevalence of emaciation, there were no statistically significant differences between grades in all years except 2016 and 2017 (*P* > 0.017). In the present study, the difference in the prevalence of overweight was statistically significant only in 2021 between the different grades, with a higher prevalence of overweight in grades 1 ~ 3 (*P* < 0.017); the differences in the prevalence of obesity between the different grades were statistically significant in 2015, 2017, 2020 and 2021, with a higher prevalence in the lower grades than in the higher grades (all *P* < 0.017). For details, see Table 6.

**Table 6:** Nutritional status of primary and secondary school students by grade, 2014~2021 [*n*(%)]

Years			2014	2015	2016	2017	2018	2019	2020	2021	*χ*^2^_CATT_	*P*
Undernutrition	Stunting	Grade 1~3	42(7.5)a	53(10.4)a	52(8.1)a	34(5.3)a	37(5.2)a	28(3.5)a	39(4.5)a	34(4.2)a	**29.4**	**<0.001**
		Grade 4~6	42(5.5)a	39(5.5)b	54(5.6)a	30(4.0)a	34(4.1)a	48(5.4)a	45(4.2)a	20(2.1)b	**12.3**	**<0.001**
		Grade 7~9	15(2.6)b	21(3.6)b	24(3.0)b	13(1.6)b	11(1.4)b	11(1.2)b	9(1.0)b	10(1.1)b	**20.1**	**<0.001**
		*χ*^2^	**14.4**	**22.1**	**18.1**	**15.4**	**17.9**	**23.4**	**20.8**	**19.1**		
		*P*	**0.001**	**<0.001**	**<0.001**	**<0.001**	**<0.001**	**<0.001**	**<0.001**	**<0.001**		
	Wasting	Grade 1~3	153(27.1)	96(18.8)	166(25.9)a	155(24.1)a	171(24.0)	170(21.28)	196(22.6)	157(19.6)	**6.2**	**0.01**
		Grade 4~6	185(24.3)	160(22.6)	198(20.6)b	133(17.9)b	173(20.7)	159(17.7)	234(22.0)	199(20.6)	2.6	0.1
		Grade 7~9	124(21.1)	122(21.1)	125(15.7)c	166(20.4)a,b	168(20.8)	167(18.7)	165(18.8)	177(19.1)	0.4	0.5
		*χ*^2^	5.8	2.7	**22.8**	**8.1**	3.1	3.8	4.3	0.7		
		*P*	0.1	0.3	**<0.001**	**0.02**	0.2	0.2	0.1	0.696		
	Total	Grade 1~3	195(34.6)a	149(29.1)	218(34.0)a	189(29.4)a	208(29.2)a	198(24.8)	235(27.1)a	191(23.9)	**26.2**	**<0.001**
		Grade 4~6	227(29.8)a	199(28.1)	252(26.2)b	163(21.9)b	207(24.7)a,b	207(23.1)	279(26.2)a	219(22.7)	**10.1**	**0.001**
		Grade 7~9	139(23.6)b	143(24.7)	149(18.7)c	179(22.0)b	179(22.2)b	178(19.9)	174(19.9)b	187(20.2)	**4.2**	**0.04**
		*χ*^2^	**16.8**	3.1	**43.5**	**13.6**	**10.1**	5.9	**14.5**	3.6		
		*P*	**<0.001**	0.2	**<0.001**	**0.001**	**0.007**	0.1	**0.001**	0.2		
Overweight		Grade 1~3	10(1.8)	19(3.7)	30(4.7)	38(5.9)	23(3.2)	43(5.4)	47(5.4)	59(7.4)a	**18.9**	**<0.001**
		Grade 4~6	22(2.9)	24(3.4)	44(4.6)	36(4.8)	43(5.1)	45(5.0)	46(4.3)	45(4.7)b	3.4	0.1
		Grade 7~9	15(2.6)	19(3.3)	35(4.4)	36(4.4)	39(4.8)	58(6.5)	31(3.5)	39(4.2)b	2.8	0.1
		*χ*^2^	1.7	0.2	0.1	1.7	3.7	2.0	3.7	**9.7**		
		*P*	0.4	0.9	1.0	0.4	0.2	0.4	0.2	**0.008**		
Obesity		Grade 1~3	10(1.8)	20(3.9)a	18(2.8)	26(4.0)a	17(2.4)	21(2.6)	40(4.6)a	54(6.7)a	**16.7**	**<0.001**
		Grade 4~6	5(0.7)	10(1.4)b	24(2.5)	24(3.2)a	27(3.2)	30(3.3)	43(4.0)a	48(5.0)a	**35.9**	**<0.001**
		Grade 7~9	10(1.7)	12(2.1)a,b	15(1.9)	9(1.1)b	19(2.4)	19(2.1)	17(1.9)b	7(0.8)b	1.0	0.33
		*χ*^2^	4.2	**8.4**	1.4	**13.1**	1.5	2.5	**10.2**	**42.9**		
		*P*	0.1	**0.02**	0.5	**0.001**	0.5	0.3	**0.006**	**<0.001**		

a, b indicate two-by-two comparisons of student frequencies for different grades with the same nutritional status. “a vs. b”, “a vs. a, b” and “b vs. a, b” indicate statistically significant differences (Bonferroni correction, *P*<0.017). “a vs. a” and “b vs. b” indicate that the differences are not statistically significant (Bonferroni correction, *P*>0.017)

### Nutritional status and changing trends of primary and secondary school students by ethnics from 2014 to 2021

From 2014 to 2021, among the school students surveyed, only Li students showed a significant decreasing trend (*P* < 0.05) in the prevalence of undernutrition, with a decrease of 8.97%, in which the prevalence of stunting and emaciation decreased by 3.27% and 5.70%, respectively, both showing a significant downward trend (both *P* < 0.05). The prevalence of overweight among Li as well as other ethnic minority students showed an increasing trend (both *P* < 0.05), with an increase of 2.70% and 5.63%, respectively. The prevalence of obesity among Han, Li, and other ethnic minorities all showed an upward trend (all *P* < 0.05), increasing by 3.51%, 2.59%, and 3.03%, respectively.

Over the 8 years, except 2020, there were statistically significant differences in the prevalence of undernutrition among different ethnic groups and was higher for the Li than for the Han (all *P* < 0.017). The differences between other ethnic minorities and the Li and Han were not significant except a few years (all *P* > 0.017). The prevalence of stunting was statistically significant between different ethnic groups in all years except 2017–2019, and the prevalence of stunting was higher in other ethnic minority students (all *P* < 0.017). The difference in the prevalence of emaciation among different ethnic groups was the same as the case of the prevalence of undernutrition. The differences in the prevalence of overweight in different years were statistically significant among different ethnic groups, all of which were higher for Han than for Li (all *P* < 0.017), and the differences between ethnic minorities and Li and Han were not significant except in individual years (*P* > 0.017). The prevalence of obesity was higher among Han than Li in 2016–2020 (all *P* < 0.017), and the difference was not significant between other ethnic minorities and Li and Han (both *P* > 0.017). For details, see Table 7.

**Table 7:** Nutritional status of primary and secondary school students of different ethnics , 2014~2021 [*n*(%)]

Years			2014	2015	2016	2017	2018	2019	2020	2021	*χ*^2^_CATT_	*P*
Undernutrition	Stunting	Han	15(3.3)a	16(3.9)a	18(3.8)a	6 (1.6)	13(3.0)	12 (2.9)	14(2.9)a	14(2.8)a,b	1.0	0.3
		Li	64(5.2)a,b	75(6.5)a,b	89(5.3)a,b	60 (3.8)	61 (3.6)	62 (3.2)	59(2.9)a	38(1.9)b	**57.0**	**<0.001**
		Other ethnic minorities	20(8.7)b	22(9.7)b	23(8.9)b	11 (4.5)	8 (3.5)	13 (5.4)	20(7.3)b	12(5.9)a	**3.9**	**0.047**
		*χ*^2^	**9.0**	**8.6**	**8.3**	5.6	0.5	3.5	**15.3**	**47.9**		
		*P*	**0.01**	**0.01**	**0.02**	0.06	0.8	0.2	**<0.001**	**<0.001**		
	Wasting	Han	81(17.9)a	60(14.6)a	77(16.4)a	58(15.0)a	75(17.1)a	60(14.4)a	91(18.5)	70(13.8)a	0.4	0.5
		Li	337(27.3)b	285(24.5)b	366(21.9)b	343(21.9)b	386(22.8)b	402(20.3)b	449(22.0)	428(21.6)b	**12.8**	**<0.001**
		Other ethnic minorities	44(19.1)a	33(14.6)a	46(17.7)a,b	53(21.5)a,b	51(22.4)a,b	34(14.1)a	55(20.2)	35(17.1)a,b	0.02	0.9
		*χ*^2^	**19.7**	**24.4**	**8.3**	**9.2**	**7.0**	**13.8**	3.1	**16.6**		
		*P*	**<0.001**	**<0.001**	**0.02**	**0.01**	**0.03**	**0.001**	0.2	**<0.001**		
	Total	Han	96(21.2)a	76(18.5)a	95(20.2)a	64(16.5)a	88(20.0)a	72(17.2)a	103(21.3)	84(16.6)a	1.1	0.3
		Li	401(32.5)b	360(31.0)b	455(27.3)b	403(25.7)b	447(26.5)b	464(24.0)b	508(24.9)	466(23.6)b	**44.7**	**<0.001**
		Other ethnic minorities	64(27.8)a,b	55(24.3)a,b	69(26.5)a,b	64(25.9)b	59(25.9)a,b	47(19.4)a,b	75(27.5)	47(22.9)a,b	1.0	0.3
		*χ*^2^	**20.6**	**25.0**	**9.6**	**14.8**	**7.8**	**10.6**	4.1	**11.5**		
		*P*	**<0.001**	**<0.001**	**0.008**	**0.001**	**0.02**	**0.005**	0.1	**0.003**		
Overweight		Han	21(4.7)a	27(6.6)a	35(7.5)a	32(8.3)a	35(8.0)a	32(7.7)a	30(6.1)	40(7.9)a	1.7	0.2
		Li	21(1.7)b	28(2.4)b	57(3.4)b	67(4.3)b	57(3.4)b	93(4.8)b	78(3.8)	87(4.4)b	**19.0**	**<0.001**
		Other ethnic minorities	5(2.2)a,b	7(3.1)a,b	17(6.5)a	11(4.5)a,b	13(5.7)a,b	21(8.7)a	16(5.9)	16(7.8)a,b	**9.2**	**0.002**
		*χ*^2^	**12.1**	**15.9**	**16.4**	**10.6**	**18.2**	**9.8**	**6.4**	**12.5**		
		*P*	**0.002**	**<0.001**	**<0.001**	**0.005**	**<0.001**	**0.007**	**0.04**	**0.002**		
Obesity		Han	10(2.2)a	15(3.7)a	21(4.5)a	23(5.9)a	22(5.0)a	22(5.3)a	31(6.3)a	29(5.7)	**9.2**	**0.002**
		Li	13(1.1)a	24(2.1)a	30(1.8)b	29(1.9)b	37(2.2)b	40(2.1)b	59(2.9)b	72(3.6)	**25.0**	**<0.001**
		Other ethnic minorities	2(0.9)a	3(1.3)a	6(2.3)a,b	7(2.8)a,b	4(1.8)a,b	8(3.3)a,b	10(3.7)a,b	8(3.9)	**6.8**	**0.009**
		*χ*^2^	3.8	4.5	**11.3**	**20.0**	**11.4**	**13.7**	**13.5**	4.5		
		*P*	0.2	0.1	**0.004**	**<0.001**	**0.003**	**0.001**	**0.001**	0.1		

“a vs. b”, “a vs. a, b” and “b vs. a, b” indicate statistically significant differences (Bonferroni correction, *P*<0.017). “a vs. a” and “b vs. b” indicate that the differences are not statistically significant (Bonferroni correction, *P*>0.017) a, b indicate two-by-two comparisons of student frequencies for different grades with the same nutritional status

## Discussion

### Improvement of the growth and development of primary and secondary students

The results of the study showed that the average height and weight of both boys and girls in Qiongzhong County were all on the rise during the 8 years of the NIPRCES, which is in line with the trend of students in other provinces in China where the NIPRCES has been implementedZhang Xiaofan [18] analyzed the changing trends of students’ growth and development levels from 2012 to 2017 in 22 central and western provinces in China implementing the NIPRCESIt can be seen that after the implementation of the NIPRCES the students’ growth and development have been improved to some extent. However, the improvement effect of students in Qiongzhong County, Hainan province was less than that in central China and even less than that in western China. This is probably due to Qiongzhong County of Hainan Province is located in a mountainous area and inhabited by ethnic minorities. Its economic and cultural levels and transportation convenience are lower than in central China and even lower than in western China. And the lower socio-economic and cultural levels will hinder the growth and development of children and adolescents [19, 20]. In addition, due to the distinctive dietary patterns of each ethnic minority group, it is difficult to completely incorporate their dietary features during the school feeding process, and the absence of nutrition and health education will reduce the efficacy of nutrition improvement. This study showed that the rate of growth in height and weight slowed down as the program was extended. The reasons may be that in the initial years of the project, the dietary quality of students during school was improved to a certain extent, so that their growth and development levels had been significantly enhanced from a low baseline level. However, if the dietary quality, such as the contents of quality protein, micronutrients, essential fatty acids in the diet, do not increase further as time goes on, the growth rate will slow down.

During this research project, the girls’ height and weight increased rapidly at 9 ~ 11 years old, while boys’ height and weight increased rapidly at 11 ~ 13 years old. And in the Zhang Xiaofan’s study, the results revealed that the growth spurt age of height and weight for girls and boys were 9 ~ 11 and 11 ~ 13 years old, respectively. But Zhang Yihong [21] reported that in 2014, the height growth peaks of Chinese adolescents were at ages 13 years old for boys and 11 years old for girls. Obviously, girls enter puberty earlier than boys, and the adolescent puberty has an earlier trend. However, entering adolescence too early is disadvantages to both the physical and mental health of adolescents [22]. Therefore, we should pay attention to the phenomenon of early puberty age, while implementing the NIPRCES to improve students’ growth and development.

### The prevalence of undernutrition among primary and secondary school students

The findings of the current study indicate a decrease in the prevalence of malnutrition among students in the areas where NIPRCES was implemented, aligning with previous research [23]. During the 8 years, the prevalences of undernutrition and wasting of the monitored students were higher than the prevalence of undernutrition (14.15% ~ 16.67%) and the prevalence of wasting (9.91% ~ 10.93%) of students who participated in the NIPRCES of other areas in China from 2012 to 2017. During the NIPRCES implementation period, the prevalence of malnutrition among local schoolchildren decreased, but it remained significantly higher than the prevalence of undernutrition among rural children aged 6 to 17 in 2012 (14.7%) as reported in the Report on Chinese Residents’ Nutrition and Chronic Disease Status (2015) [24]. It may be because Hainan is the only tropical province in China, and because of the hot weather, people have a light diet and often take porridge as their staple food and drink more water, thus reducing the total dietary energy intake. And it could also be the high temperature of the environment increasing the energy expenditure of the body. Collectively, after the implementation of the NIPRCES, the undernutrition among primary and secondary school students in Qiongzhong County, Hainan Province has been significantly improved, but the problem is still serious and the reasons need to be further explored.

It also showed that, from 2014 to 2021, the prevalences of undernutrition and wasting for boys were higher than that for girls, and the prevalence of stunting was higher among boys than girls except a few years, consistent with the results of other relevant studies [18, 23]. It could be because compared with girls, boys have faster growth rates, more energy consumption in the same exercise, and higher nutrient requirements [25, 26], so that they are more likely to suffer from undernutrition when the dietary quality is not high. This phenomenon is likely influenced by the divergent levels of nutrition knowledge and dietary behaviors between dietary choices made by boys and girls. Many studies have shown that compared with girls, boys have lower nutrition knowledge and more bad dietary behaviors [27].

During the 8 years, the prevalences of stunting for primary school students (grades 1 ~ 3 and grades 4 ~ 6) were both significantly higher than that for middle school students (grades 7 ~ 9). The reasons may be that compared with middle school students, primary school students have higher basal metabolic rate, more physical activity, and higher nutrient requirements [28]. So, the unreasonable diet at this stage is more likely to lead to undernutrition. The prevalence of stunting among primary school students was high, indicating that a large number of primary school students have long-term nutritional deficiencies. The reasons may be that students’ meals are mainly provided by families before entering school and at primary school, while parents in poverty-stricken areas have lower health literacy, lack nutritional knowledge, and have a weak awareness of reasonable diets, which lead to long-term unreasonable dietary structure for children, then the prevalence of stunting is high [29].

The subjects monitored in this study were derived from ethnic minority autonomous counties and analyzed in three groups of Li, Han, and other ethnic minorities (mainly including Miao, Zhuang, and Yao). It revealed that the prevalences of undernutrition and wasting were greater among Li than Han in 8 years (except for 2020), which is consistent with Wang Zeyuan’s [30] findings and suggested this may be due to the genetic, dietary patterns, lifestyle, economic, and cultural level variables [31, 32].Scholars have analyzed the body composition and shape of the Li and discovered that they are thinner and have more developed muscles than Han [33].

### The prevalence of overweight and obesity in primary and secondary school students

The results show an increasing trend in monitoring the prevalence of overweight and obesity among students from 2014–2021, consistent with the findings of Zhang Xiaofan [18] and Hu Xiao [34]. The prevalence of overweight among the monitored students in this study was significantly lower than the prevalence of overweight among students who participated in the NIPRCES of other provinces in China from 2012 to 2017 [18]. However, the prevalence of obesity among students in 2021 in this study (4.05%) was significantly higher than that in the other areas in China 2017 monitoring results (3.74%) [18]. Of course, the prevalences of overweight and obesity were still significantly lower than the prevalence of overweight (11.1%) and the prevalence of obesity (7.9%) among Chinese children and adolescents aged 6 to 17 reported in the Report on Chinese Residents’ Nutrition and Chronic Disease Status(2020) [35]. The prevalence of overweight and obesity among students monitored under NIPRCES in Hainan showed a higher average annual rate of increase compared to that in other provinces. The authors considered that the reasons for this might be the low baseline levels of overweight and obesity among the subjects monitored in this study. With improved food access and local economic development, the food intake of primary and secondary school students was no longer excessively limited by economic conditions, which leaded to a rapid increase in the prevalences of overweight and obesity.

The results showed that the prevalences of overweight and obesity among monitored students were higher among boys than girls from 2014 to 2021, which were consistent with the results of several studies in China and abroad [36–38]It may be related to the physiological variations between boys and girls, their food habits, and preoccupation with body shape. Compared with girls, boys usually eat more and prefer high-energy foods such as meat and carbonated drinks. In addition, boys are more likely to be less physically active and less conscious about their body shape because they are addicted to online games [39]. The results may also be related to the existence of a more obvious patriarchal ideology in Hainan. Wang Xue Chen [40] through the gender culture behind the “Dad ’s Tea” (a special snack from Hainan)study found, there is a deep-rooted patriarchal cultural view in Hainan, and the phenomenon of “men at home and women at work” is common in family life. As a result, the boys in the family usually can access to more food resources and pocket money than the girls.

In about half of the 8 years, the prevalence of obesity among primary school students were significantly higher than that of middle school students, and the prevalence of overweight among primary school students ( grades 1 ~ 3) was significantly higher than in other grades. The reasons for this may be that, for primary school students, except for the lunch at school, the rest meals are provided by their families. However, parents in rural areas have low literacy and lack the concepts of reasonable meals. Especially when economic conditions improve, students may be provided with a great deal of meat and other foods that parents believe to be high in nutritional value but also high in energy, and even given more snacks with high energy density and low nutrient density according to student preferences. The primary students’ self-cognition ability and autonomous action ability are low, making it difficult to consciously form healthy dietary behaviors. When combined with the fact that students, especially students in low grades, have not yet reached the age of pursuing external beauty and caring about body shape. These are likely to cause excess dietary energy intake, resulting in overweight and obesity.

Comparing different ethnic groups over a period of 8 years, this study found that the prevalences of overweight and obesity were greater among Han than Li, which may be because Li students are mostly from more remote rural areas, while Han Chinese students are mostly from town areas where food sources are more abundant and accessible. This may also be because compared with the Li, Han families generally have fewer children or even only children, and the economic conditions are better, which make it easier for Han students to obtain foods, including high-energy snacks, in addition, they have less physical activities. All these may contribute to higher prevalences of overweight and obesity among Han Chinese students than among Li.

Drawing from the aforementioned findings, future studies could focus on addressing the nutritional enhancement requirements of students from various ethnic backgrounds within the same region. Furthermore, researchers can delve into factors that contribute to shifts in the nutritional status of student population following their participation in the Nutrition Improvement Program.

## Suggestion

The high prevalence of undernutrition has been a persistent issue among students in compulsory education in the region, while overweight and obesity have emerged as growing concerns in recent years. This study highlights that despite implementing the same nutritional improvement program in the same area, there are still notable differences in the trend of nutritional improvement among students based on gender, ethnicity, and age.

Therefore, in the follow-up implementation of the NIPRCES in Hainan Province, we should pay high attention to the coexistence of undernutrition and overnutrition among students, integrate nutrition and health education into the NIPRCES, strengthen nutrition education for students, teachers and parents, and guide students to form scientific dietary and exercise habits. At the same time, the dietary characteristics and food availability in areas where ethnic minorities live should be fully considered in school meals, so as to provide reasonably priced, nutritionally balanced and delicious meals.

## Strengths and limitations

There are several strengths to this study. It effectively illustrates the trend in the nutritional status of primary and secondary school students participating in the NIPRCES in Qiongzhong County, Hainan Province from 2014 to 2021. Furthermore, the study also examines the variations in the trend of changing nutritional status among students of different ethnic groups within areas that have implemented similar nutritional improvement measures. This study furnishes a substantial amount of foundational data to bolster the continuous monitoring of the impacts of NIPRCES implementation in Hainan Province. Furthermore, it sets the groundwork for future research endeavors aimed at investigating additional factors influencing the nutritional status of students in the region.

Nonetheless, this study still has some limitations. Firstly, although this study was conducted as a cohort study there were still unobserved variables that could have influenced the outcome, so this study is still restricted in terms of causal inference. Secondly, this study did strict quality control based on the existing material, but it is still difficult to avoid the change of meal delivery policy or the subjective non-consumption of nutritious meals by students. Due to the length of the article, this study does not discuss the situation before and after the change of the meal delivery policy. Finally, this study incorporated existing confounders such as gender and ethnicity as subgroup variables in the subgroup analyses as much as possible to reduce the impact of confounding on the results. However, it was impossible to ensure that all confounding variables were included in the subgroup variables.

## Conclusions

During the 8 years of NIPRCES implementation in Qiongzhong County, Hainan Province, the growth level and undernutrition of primary and secondary school students have been improved. However, both the aggregate level and the rate of improvement are lower than the national level. Although the prevalence of undernutrition is declining, it is still high and is mainly wasting. At the same time, the prevalences of overweight and obesity are on the rise. The problem of both undernutrition and overweight obesity was more prominent among boys and lower-grade students, that is, they face more severe double burden of undernutrition and over-nutrition. Besides, compared with students of other ethnic groups, the problem of undernutrition among Li students is more pronounced, while the problem of overweight and obese among Han students are more prominent.

## Data Availability

The datasets generated and/or analysed during the current study are not publicly available due to our original data contains detailed personally identifiable information, we are unable to provide you with the original data in order to keep the promises we made to our respondents in the informed consent form,but are available from the corresponding author on reasonable request.
